# *Mycobacterium tuberculosis* and SARS-CoV-2 Coinfections: A Review

**DOI:** 10.3389/fmicb.2021.747827

**Published:** 2022-02-03

**Authors:** Narjess Bostanghadiri, Faramarz Masjedian Jazi, Shabnam Razavi, Lanfranco Fattorini, Davood Darban-Sarokhalil

**Affiliations:** ^1^Department of Microbiology, School of Medicine, Iran University of Medical Sciences, Tehran, Iran; ^2^Microbial Biotechnology Research Center, Iran University of Medical Sciences, Tehran, Iran; ^3^Department of Infectious Diseases, Istituto Superiore di Sanità, Rome, Italy

**Keywords:** *Mycobacterium tuberculosis*, TB, COVID-19, SARS-CoV2, coronavirus, coinfection

## Abstract

**Background:**

Tuberculosis (TB) is still one of the most important causes of death worldwide. The lack of timely attention on TB diagnosis and treatment during the coronavirus disease 2019 (COVID-19) pandemic is a potential threat to health issues and may have severe consequences for patients and health systems. There is not much information on the management of TB during this period. Here, we reviewed the current literature to evaluate the rate of *Mycobacterium tuberculosis* and severe acute respiratory syndrome coronavirus 2 coinfections and interactions between these infectious agents.

**Methods:**

Several databases, including Web of Science, Scopus, and MEDLINE (via PubMed), were searched for original articles addressing TB and COVID-19 diseases published from December 2019 to April 2021.

**Results:**

Of 3,879 articles, 57 articles were included in this study, and among 106,033 patients affected by COVID-19, 891 also had TB. Overall, investigators found a consistent increase in C-reactive protein, D-dimer (especially in patients with severe clinical manifestation), erythrocyte sedimentation rate, lactate dehydrogenase, alanine aminotransferase, and a reduction of lymphocytes. The respiratory symptoms of TB/COVID-19 patients were similar to those of TB patients, but the risk of developing pulmonary TB increased in COVID-19 patients. Also, the mortality rate in TB/COVID-19 patients was higher than that in patients affected only by COVID-19 or TB.

**Conclusion:**

Some reports indicated worsening respiratory symptoms and even activation of latent TB after COVID-19 or vice versa. It seems that both active and previously treated TB constituted a risk factor for COVID-19 in terms of severity and mortality, regardless of other underlying diseases and patient status. Health systems should not neglect TB during this era of the ongoing COVID-19 pandemic by setting up appropriate diagnostic and clinical management algorithms.

## Introduction

Tuberculosis (TB) is an infectious disease that seriously affects human health. *Mycobacterium tuberculosis* (MTB) infects the lungs primarily, although it may also spread to other organs and tissues, including intestines, liver, lymph nodes, skin, brain, and musculoskeletal and reproductive systems (Mbuh et al., 2019; Mathiasen et al., 2020).

It is estimated that one-quarter of the world’s population is latently infected with MTB, 5–15% of whom develop active TB in their lifetime; the reactivation risk varies geographically and individually (Mousquer et al., 2020). The emergence of multidrug-resistant (MDR) and extensively drug-resistant (XDR) TB has complicated the control of the disease due to the need for hospitalization and expensive medications. Recently, resistance to the new drugs, bedaquiline and delamanid, has been reported in MDR/XDR MTB strains, making it difficult to treat the disease caused by these organisms (Schrager et al., 2020).

The coronavirus disease 2019 (COVID-19), caused by the severe acute respiratory syndrome coronavirus 2 (SARS-CoV2), was identified in December 2019 in Wuhan, China, and promulgated as a pandemic by the World Health Organization (WHO) on March 11, 2020. The COVID-19 pandemic has become a great public health threat by the exponential expansion worldwide with considerable impact on morbidity and mortality and economic disruption (Carlos et al., 2020; Wang C. et al., 2020). Approximately 80% of COVID-19 patients demonstrated mild to moderate symptoms, and 20% showed severe disease (Liu K. et al., 2020). TB and COVID-19 affect the lungs, interfere with the host immune system, and have similar symptoms. The most common clinical manifestations of COVID-19 are fever, respiratory symptoms, and cough, whereas less severe symptoms are fatigue, headache, myalgia, hemoptysis, and gastrointestinal (GI) symptoms such as vomiting and diarrhea (Lin et al., 2020; Yang et al., 2020). Prolonged cough is the main symptom of TB and COVID-19. Other TB symptoms are bloody sputum accompanied by fever, hemoptysis, dyspnea, night sweats, appetite loss, and weight loss (Wei et al., 2020). Limited information is available on the risk of disease or severe consequences in patients with TB and COVID-19. However, previous studies have suggested the exacerbation of TB in coinfection with some viruses, such as measles (Yang and Lu, 2020). TB patients are at risk of coinfection with COVID-19 (Fattorini et al., 2020; Jiménez and Cevallos, 2021), although its effect on mortality requires further investigation. Because of similar symptoms between the two diseases, the WHO Global TB control programs faced serious challenges, impairing TB diagnosis and treatment and neglecting TB among COVID-19 patients, leading to the increase in drug-resistant MTB strains. Moreover, both TB and COVID-19 have similar risk factors including immune system defects, diabetes, poverty, overcrowding, and air pollution (Getnet et al., 2019; Mousquer et al., 2020; Togun et al., 2020).

Despite studies attempting to elucidate interactions between MTB and SARS-CoV-2, several uncertainties remain. Conducting a comprehensive survey on different aspects of MTB and SARS-CoV-2 coinfections, including incidence, mortality, clinical manifestation, diagnosis, treatment, and laboratory evidence, may be helpful for the management of both diseases. Thus, we explored updated literature on MTB and SARS-CoV-2 coinfections reported in 2020–2021.

## Materials and Methods

This review was performed according to current guidelines, and results reported conforming to the PRISMA (Preferred Reporting Items for Systematic Reviews and Meta-Analyses) extension for scoping review (Peters et al., 2015; Hay-Smith et al., 2016).

### Search Strategy and Exclusion Criteria

We searched eligible studies published from December 2019 to April 2021 through PubMed, Scopus, and Web of Science. The following search terms/keywords were used: [“COVID-19” OR “SARS-CoV-2” OR “Coronavirus” OR “COVID 19” OR “2019-nCoV” OR “2019 nCoV” OR “SARS CoV 2”] AND [“*Mycobacterium tuberculosis*” OR “mycobacterial infections” OR “TB” OR “tuberculosis”]. We used Mesh, EMtree, and the free text method to determine synonyms. Relevant studies were reviewed for titles and abstracts to exclude irrelevant abstracts referring to conferences, narrative reviews, animal studies, *in vitro* studies, studies without interventions or exposures of interest, irrelevant news and letters, and duplicate studies. We checked titles and abstracts with inclusion and exclusion criteria. After the screening, eligible studies were downloaded, and the reviewers further read the full texts. After that, reviewers reached a consensus on which studies would be included for review.

### Inclusion Criteria

Inclusion criteria were studies published without language limitation that established co-occurrence of COVID-19 and TB. Study designs included case studies, case series, original studies, and observational studies.

Studies reporting COVID-19 coinfection without TB, those reporting other outcomes, letters to the editor, and theoretical and incomplete studies were excluded.

## Results and Discussion

### Search Results

**FIGURE 1:**
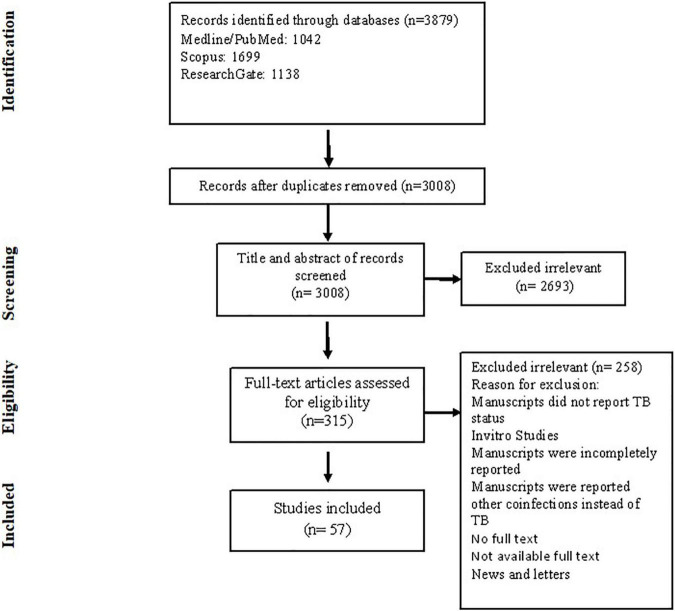
Flowchart of literature search and study selection process.

**FIGURE 2:**
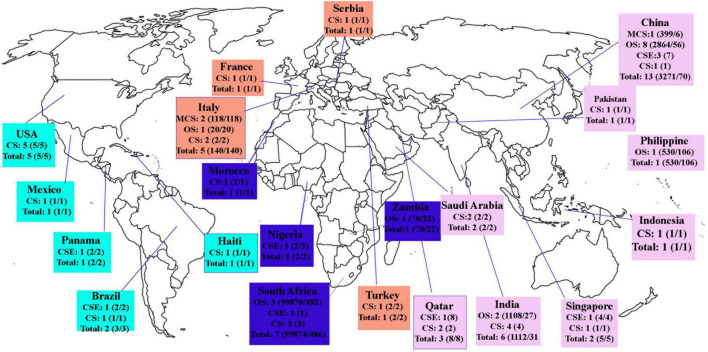
Studies conducted among TB/COVID-19 patients in different countries. MCS, multicenter studies; CSE, case series; CS, case studies; Total, number of patients with COVID-19/TB.

Electronic combined search terms identified 3,879 articles. Inclusions and exclusions were reported following PRISMA guidelines presented in a PRISMA flow diagram (Figure 1) with reasons for exclusion recorded as follows: 871 duplicates were removed; after reading the titles and abstracts, 2,693 articles were removed. Three hundred fifteen full-text studies were assessed for eligibility, and 258 were excluded because of incomplete, irrelevant, or unavailable full text. For analysis, 57 studies included 28 case studies, 10 case series, and 19 original studies performed in different continents (Figure 2). Each article meeting inclusion criteria was substantially assessed for the author, country, year, cross-section, type of study, age, gender, number of COVID-19 cases, number of TB patients, TB and COVID-19 diagnostic methods, laboratory criteria, clinical features, MTB types, treatment of TB and COVID-19, recovery, and mortality rate.

Based on our findings, **Tables 1, 2** summarize original studies, case studies, and case series, respectively. The global estimated incidence of TB was 130 cases per 100,000 population in 2019 (TBFACTS, 2019). Our literature review found 106,033 COVID-19 cases, including 891 cases with TB coinfection, indicating high TB incidence in COVID-19 patients compared with global estimated incidence. Most data in case studies and case series were for adults (range of median ages, 22–77 years), with only three studies reporting data exclusively from children (Essajee et al., 2020; Goussard et al., 2020a,b). Overall, in these studies, males accounted for 41 (75%) of TB/COVID-19 coinfection. The median age in original studies ranged from 36 to 66 years, in which males showed higher MTB and SARS-CoV-2 coinfections compared with females. One study included data from children only, with a median age of 48 months (van der Zalm et al., 2020).

### Methods Used for Tuberculosis and COVID-19 Diagnosis

A wide range of diagnostic tests is available for TB and COVID-19. Standard techniques (smear microscopy and culture methods) and WHO-approved molecular tests (automated cartridge-based nucleic acid amplification assays such as GeneXpert, Xpert MTB/RIF, Xpert Ultra, Truenat test) were used for TB diagnosis (Liu Y. et al., 2020; WHO, 2020). Almost all studies used real-time reverse transcription–polymerase chain reaction (RT-PCR) assays to search SARS-CoV-2 in nasopharyngeal and throat swabs, blood, sputum, and bronchoalveolar lavage (Chen et al., 2020; Ferrari et al., 2020; Liu J. et al., 2020; Liu Y. et al., 2020; Sy et al., 2020; van der Zalm et al., 2020; Wang W. et al., 2020; Zhang et al., 2020; Goel et al., 2021; Li et al., 2021; Mwananyanda et al., 2021; Wang et al., 2021). The sensitivity of RT-PCR as a potential diagnostic tool was between 66 and 80%, depending on virus load in the patient (Jiménez and Cevallos, 2021). Identifying specific genes was applied by RT–quantitative PCR or RT loop-mediated isothermal amplification, with a sensitivity of 95% (Corman et al., 2020; Udugama et al., 2020).

### Laboratory Criteria

The specific SARS-CoV2 response can be detected in the whole blood in the acute phase and the convalescents (Petrone et al., 2021). Blood test analysis showed a relevant increase of C-reactive protein (CRP), D-dimer, erythrocyte sedimentation rate, lactate dehydrogenase, alanine aminotransferase (ALT), procalcitonin, ferritin, fibrinogen. Less common abnormalities were elevated concentrations of random blood glucose, adenosine deaminase, ALT, aspartate aminotransferase, creatine phosphokinase, platelet count, NT-proB-type natriuretic peptide, total bilirubin, conjugated bilirubin, blood urea nitrogen, red cell distribution width, and creatinine. Decreased red blood cell count, hemoglobin, hematocrit, mean corpuscular hemoglobin concentration, and serum albumin were also reported (Li et al., 2020; Singh et al., 2020; van der Zalm et al., 2020; Hesse et al., 2021). Some factors such as D-dimer and CRP were significantly higher in patients with severe diseases (Wang et al., 2021). In most studies, leukopenia, particularly lymphocytopenia, was reported. In contrast, neutrophilic leukocytosis was observed in some studies (Garg and Im Lee, 2020; Liu J. et al., 2020; Singh et al., 2020; Mishra et al., 2021; Stjepanović et al., 2021). Two studies from China (Wuhan and Sichuan) found that most SARS-CoV-2 patients had lymphopenia and eosinopenia (Wang et al., 2021). In other studies, a high concentration of serum amyloid A was found (Zhang et al., 2020). In general, COVID-19 cases with severe clinical manifestations had more abnormalities, including elevated serum levels of interleukin 2 (IL-2), IL-2R, IL-6, IL-10, IL-7, tumor necrosis factor, and low cellular immunity (e.g., CD3^+^, CD3^+^CD4^+^, CD3^+^CD8^+^) (Garg and Im Lee, 2020; Liu C. et al., 2020; Mishra et al., 2020).

### Clinical Features of Tuberculosis/COVID-19 Coinfections

COVID-19 manifestations (asymptomatic, mild, severe, fatal) included respiratory and non-respiratory symptoms. Significant clinical symptoms of COVID-19 were fever, dry cough, and dyspnea. Other non-specific symptoms included fatigue, myalgia, headache, and GI symptoms, such as anorexia, nausea, vomiting, diarrhea, abdominal pain, discomfort, and GI bleeding. The severity of the disease and symptoms varied from person to person, so that some patients were transferred to the intensive care unit after hospitalization. Respiratory symptoms were mostly similar to those of TB.

Tuberculosis and COVID-19 show similarities that may make the diagnosis difficult; however, the onset of TB symptoms was usually gradual, and duration was longer than that of COVID-19, lasting from weeks to months. Pulmonary TB patients presented with fever, productive cough, night sweating, weight loss, hemoptysis, dyspnea, fatigue, and loss of appetite (Shanmuganathan and Shanmuganathan, 2015). Some people with COVID-19 were referred to the hospital with characteristic TB symptoms, such as fever, night sweats, weight loss, reduced appetite, and cough with occasional blood-streaked sputum, and further evaluations and tests confirmed the simultaneous presence of MTB and SARS-CoV-2 organisms (Cutler et al., 2020; Gbenga et al., 2020; Singh et al., 2020; Tham et al., 2020; Yadav and Rawal, 2020; Baskara et al., 2021; Musso et al., 2021; Pillay and Magula, 2021). As lung damage caused by TB increases the body’s susceptibility to getting infected with other airborne infections, it can be considered that TB can be a risk factor for exacerbating the severity of COVID-19 patients (Mousquer et al., 2020). Moreover, impairment of the immune mechanism and cytokine overexpression play a vital role in TB exacerbation (Crisan-Dabija et al., 2020; Visca et al., 2021). Therefore, COVID-19 may be a predisposing factor for the conversion of latent TB to active TB and worsening of COVID-19 severity and progression of TB (Parseh et al., 2021).

### Pulmonary and Extrapulmonary Tuberculosis in COVID-19

Gupta et al. examined 22 patients with TB and COVID-19. Of these, 13 had active TB, and nine were previously treated TB cases. Among those with active TB, nine had pulmonary TB and four extrapulmonary TB, including cerebral tuberculoma, pleural effusion, cervical lymphadenopathy, and disseminated TB (Gupta et al., 2020). Other cases of COVID-19 in patients with active pulmonary and/or extrapulmonary TB were reported. In Italy, Stochino et al. (2020) described renal and neurological TB, and Tadolini et al. (2020) performed a large multicenter observational study including COVID-19 patients with pulmonary and/or extrapulmonary TB. Extrapulmonary TB involved bone, larynx, central nervous system, lymph nodes, peritoneal, GI, genitourinary, pleural, and spinal tissues (Tadolini et al., 2020). Also, miliary TB was reported in COVID-19 patients (AlKhateeb et al., 2020; Bouaré et al., 2020; Elziny et al., 2021). Several other studies described pulmonary and extrapulmonary TB cases, with the former being the most prevalent (AlKhateeb et al., 2020; Cao et al., 2020; Goussard et al., 2020a,b; He et al., 2020; Liu C. et al., 2020; Sarma et al., 2020; Sasson et al., 2020; Singh et al., 2020; Yao et al., 2020; Yousaf et al., 2020; Ayad et al., 2021; Musso et al., 2021).

### Treatment of Tuberculosis and COVID-19 Diseases

No specific treatments for COVID-19 have been discovered so far (WHO, 2021a). Various drugs were used for treating COVID-19 patients with or without TB, including protease inhibitors (PIs) (atazanavir, lopinavir/ritonavir, darunavir, cobicistat, raltegravir), nucleoside analogs, antiviral agents (remdesivir, ribavirin) preventing viral membrane fusion (umifenovir or arbidol), antimalarial agents (chloroquine, hydroxychloroquine [HCQ]), non-specific anti-inflammatory drugs (methylprednisolone, dexamethasone), anticoagulants, and immunosuppressive drugs. Antibiotics, such as azithromycin, moxifloxacin, linezolid, cycloserine, clofazimine, pyrazinamide, ceftriaxone, and carrimycin (a new macrolide), were administered to these patients. Tadolini et al. (2020) reported 49 patients with active/previously treated TB and COVID-19 infections in eight countries. Patients received first- and second-line anti-TB drugs. Medications for COVID-19 included antiviral drugs (darunavir/cobicistat, lopinavir/ritonavir), antibiotics (azithromycin), and HCQ (Tadolini et al., 2020). In a large cohort study in Wuhan, investigators found that administration of ganciclovir or oseltamivir reduced the risk of death in severe patients, and glucocorticoids increased the risk of progression from non-severe to severe status (Liu J. et al., 2020). Theoretically, the combination of first-line anti-TB and some antiviral treatment for COVID-19 should have increased the risk of adverse effects (Tadolini et al., 2020). Rifampicin is the most potent inducer of multiple genes that control drug metabolism, such as cytochrome P450 isoforms, including CYPs 1A2, 2C19, 2C9, 2D6, and 3A4 (Baciewicz et al., 2013). The CYP3A4 isoenzyme extensively metabolizes PIs. Hence, coadministration of PI drugs with rifampicin reduces the systemic concentration of PIs (Acosta et al., 2007; Karanja et al., 2016). For this reason, Cao et al. (2020) used umifenovir instated of lopinavir/ritonavir. However, other cases with active pulmonary TB and COVID-19 were treated with rifampicin and lopinavir/ritonavir (Faqihi et al., 2020; Luciani et al., 2020; Yao et al., 2020). In addition, rifampin can decline the effectiveness of chloroquine by inducing powerful enzymes (Bouaré et al., 2020). According to the results, the use of immunosuppressive drugs in the treatment regimen of patients with COVID-19 may increase the risk of active TB due to reactivation or new MTB infection, even in later cases of pandemic (Minozzi et al., 2016; Yang and Lu, 2020). Because of the incompleteness of the treatment process in most patients, there are no sufficient data to understand the potential effect of COVID-19 on the TB treatment and outcome.

### Comorbidity

Underlying diseases reported in TB/SARS-CoV-2 coinfections were diabetes mellitus, hypertension, hypothyroidism, seizure disorder, renal cancer, cardiac disease, asthma, HIV/AIDS, chronic obstructive pulmonary disease, prostate cancer, liver cirrhosis related to hepatitis B and D viruses, and atrial fibrillation (Bouaré et al., 2020; Cao et al., 2020; Farias et al., 2020; Faqihi et al., 2020; Garg and Im Lee, 2020; Gupta et al., 2020; Motta et al., 2020; Orozco et al., 2020; Pinheiro et al., 2020; Rivas et al., 2020; Sarınoğlu et al., 2020; Sasson et al., 2020; Sy et al., 2020; Tadolini et al., 2020; Baskara et al., 2021). In general, older patients with comorbidities were more susceptible to COVID-19 (Huang et al., 2020). Chen et al. (2020) showed that active or latent TB increased susceptibility to COVID-19 and disease severity; however, more extensive studies are necessary (Liu Y. et al., 2020).

### Recovery and Mortality Rate

Globally, as of October 20, 2021, there were 241,411,380 confirmed COVID-19 cases, including 4,912,112 deaths (approximately 2.2%) reported to WHO (WHO, 2021b). Case fatality rates in TB/COVID-19–coinfected patients in studies by Sy et al. (2020; Wang et al., 2021), Gupta et al. (2020; Wang W. et al., 2020), Tadolini et al. (2020; Musso et al., 2021), and Motta et al. (2020; Baskara et al., 2021) were 27.3, 12.3, 11.6, and 23.6%, respectively, which were values higher than those in COVID-19 patients alone. In Lusaka (Zambia), the five most common comorbidities observed among people who died with COVID-19 were TB, hypertension, HIV/AIDS, alcohol misuse, and diabetes. Approximately 15% of all participants who died had COVID-19 (Mwananyanda et al., 2021). However, Mash et al. (2021) found that in TB, structural lung disease was not associated with increased mortality. Instead, Mishra et al. (2021) found that old age (>70 years) and 2 or more medical comorbidities contributed to mortality. Similarly, in a single-center retrospective study performed in Wuhan, older patients with chronic comorbidities had a high mortality rate (34.5%) (Chen et al., 2020). Du et al. (2020) identified other risk factors for high mortality among COVID-19 patients, including preexisting simultaneous cardiovascular or cerebrovascular diseases, age ≥ 65 years, CD3^+^ and CD8^+^ T cells ≤ 75 cells ⋅ μL^–1^, and cardiac troponin I ≥ 0.05 ng ⋅ mL^–1^ (Essajee et al., 2020). In this study, of 179 SARS-CoV-2–positive patients, eight were TB patients, all of whom survived (Du et al., 2020). Overall, mortality rates for COVID-19 among non-severe and severe patients were estimated to be 1.1 and 32.5%, respectively (Li et al., 2020). Therefore, TB can increase the mortality rate in COVID-19 patients, but further studies are required despite these contradictions.

### Limitations

Almost all included studies were observational retrospective investigations and had several limitations. Some of the primary studies had poor methodological quality and level of evidence, the most important of which was the small sample size. Furthermore, in some cases, they lacked accurate inclusion and exclusion criteria. Personal histories of patients and details on organ involvement were mainly unavailable. Changes in the laboratory results such as biochemical characteristics, inflammatory markers, and coagulation records in coinfection were not studied in this group of patients. Details of treatment such as reasons for choosing the drug; dose, duration, and side effects; and drug interactions for treatment of this subset of patients were not reported in some studies. Moreover, there are limited data on the risk of severe disease or outcomes in patients with the concurrence of TB and COVID-19. Based on these restrictions, researchers are advised to investigate these issues in their further research.

## Conclusion

Coinfections with SARS-CoV-2 and MTB are a concern and have not yet been well studied. Clinical features in symptomatic TB/COVID-19 patients are similar to those with TB, making it challenging to perform early diagnosis and treatment. MTB infection status of COVID-19 patients should be checked regularly at hospital admission. Epidemiological history and exposure, clinical manifestations, laboratory tests, and imaging examinations should be considered as comprehensive measures for rapid diagnosis, quarantine, and treatment. Present data demonstrated a direct link between COVID-19 and TB, which indirectly contribute to each other’s morbidity and mortality. Because the effects of SARS-CoV-2 and immunosuppressive drugs may temporarily inhibit immune function, in the future it may result in TB reactivation. We should be aware of the increment of the TB epidemic after the pandemic of COVID-19. It requires prompt diagnosis and public awareness to deal with both diseases. Future studies should examine the impact of TB and COVID-19 coinfection in terms of morbidity and mortality.

COVID-19 put significant pressure on different parts of the healthcare systems, and many deaths are predicted for TB patients because of disruptions in TB programs, diagnostic delays, discontinuation of treatment, and lack of access to medication. Because of the ongoing spread of the COVID-19 pandemic and the high prevalence of TB worldwide, interactions and possible effects between these two infections should be considered. In most countries, the information regarding COVID-19 is not complete yet, and information on TB does not include many clinical and immunological parameters, which would be helpful to understand the interaction between the two diseases.

Prospective studies with higher quality are required to provide a more accurate understanding of the effects of these two infections on each other.

## Author Contributions

SR and DD-S conceived and designed the review and participated in writing the manuscript and identifying eligible studies. NB played a full role in writing the manuscript, identifying eligible studies, assessing studies quality, and assisting with data extraction. FMJ assisted in data extraction, assessing studies quality, and reviewing the manuscript. LF participated in critical manuscript editing. All authors approved the definitive version of the manuscript.

## Conflict of Interest

The authors declare that the research was conducted in the absence of any commercial or financial relationships that could be construed as a potential conflict of interest.

## Publisher’s Note

All claims expressed in this article are solely those of the authors and do not necessarily represent those of their affiliated organizations, or those of the publisher, the editors and the reviewers. Any product that may be evaluated in this article, or claim that may be made by its manufacturer, is not guaranteed or endorsed by the publisher.
