# Supplementary material for: Mycobacterium tuberculosis and SARS-CoV-2 Coinfections: A Review
Source: Front Microbiol. 2022 Feb 3;12:747827. doi: 10.3389/fmicb.2021.747827 (PMC8851160; doi:10.3389/fmicb.2021.747827)
Supplement: Supplementary file 1 [file Table_1.DOCX]

Table 1. COVID-19/TB original article information

| **Author** | **Country** | **Year** | **cross section** | **Type of study** | **Age** | **Gender (M/F)** | **No. of cases (covid-19)** | **No. Patients (TB)** | **Other Lab criteria** | **Clinical feature** | **Type of TB (Strain)** | **Treatment regimen (TB) (COVD-19)** | **COVID-19**  **Recovery rate/**  **Mortality rate** | **Ref.** |
| --- | --- | --- | --- | --- | --- | --- | --- | --- | --- | --- | --- | --- | --- | --- |
| Nitesh Gupta | India | 2020 | February 1 2020 to June 14 2020 | Retrospective observational | 36.44 | 20/2 | 1073 | 22 | Not mention | fever, dry cough and dyspnoea | 13 active TB,  9 treated TB | - anti-TB  - Not mention | 16 (72.7%)/ 6 (27.3%)  3 out of active TB and 3 out of treated TB | (57) |
| Marina Tadolini | Italy (multi centres) | 2020 | Not report | **Multi-centre studies** | 48 | 40/9 | 49 | 49^1^ | Not mention | Fever, dry cough, dyspnoea | 42 active TB and 7 post-TB treatment sequelae ^2^  (4 MDR TB) | - first and second line  -HCQ, lopinavir/ritonavir, darunavir/cobicistat, azithromycin | 43/6 (12.3%) | (59) |
| Yu Chen | China | 2020 | Jan 26, 2020 and Feb 15, 2020 | observational case-control | 47 | 18/18 | 36 | 11 | Increase: CRP, LDH | cough, dyspnea, fever, lymphopenia, fatigue | Active TB:3 (MDR:1) | Not mention | Not mention | (24) |
| Jiao Liu | China | 2020 | December 29, 2019, to February 28, 2020 | retrospective | 57 | 635/555 | 1190 | 15 | Increase: D-dimer | fever, cough, dyspnea,  fatigue,  sputum production | TB history:5 | 977 patients were  treated with empirical antibiotic,  681  antiviral therapy,  289 glucocorticoids | 1033 (10 TB) / 157 (5 TB) | (25) |
| TieLong Chen | China | 2020 | January 1, 2020, to February 10, 2020 | retrospective | 54 | 108/95 | 203 | 4 | Increase: CRP, LDH, D-dimer, ESR, Procalcitonin | Fever, dry cough, chest distress | Old TB:3 | Arbidol, lopinavir/ritonavi, interfron | 177/26  (12.8%)  1 TB >65Y | (26) |
| Rong-Hui Du | China | 2020 | 25 December 2019 and 7 February 2020 | prospective | 57.6±13.7 | 97/82 | 179 | 8 | Increase: CRP and procalcitonin | Fever, dry cough, Dyspno, fatigue, sputum production and headace | Not mention | Not mention | 158 (All TB se)/21(11.7%) | (86) |
| Karla Therese L | Philippines | 2020 | May 17, 2020, following them until June 15, 2020 |  | 48.9 | 373/157 | 530 | 106 | Not mention | Not mention | Not mention | Not mention | 359 (57 TB) / 71 (25 TB)^3^ | (27) |
| Guangdi Li | multi-country | 2020 | January and April 2020 | **Multi-centre studies** | 66 | 215/184 | 399 | 6 | Not mention | fever, cough, shortness of breath, and fatigue | Not mention | Not mention | 242 (3TB)/157 (3TB) | (28) |
| Jin-jin Zhang | China | 2020 | January 16 to  February 3, 2020 |  | 57 | 70/70 | 140 | 2 | Increase: CRP, serum amyloidA, D-dimer,  serum procalcitonin and creatine kinase | fever or respiratory  symptoms |  | Not mention | Not mention | (29) |
| I.Mottaa | multi-country | 2020 |  | Retrospective | 45-82 | Among 8 dead (7/1) | 69^3^ | 69 | Not mention | Not mention | Not mention | Antivirals, steroids | 61/8 (11.6%) | (77) |
| Marieke M. van | South Africa | 2020 | 17th of April_24th of July |  | 48 months | 88/71 | 159 | 2 | Increase: CRP and platelet counts | Cough, Fever, Tight chest, Diarrhea, Vomiting | Previous and active TB | Ganciclovir, oseltamivir, arbidol, lopinavir and ritonavir, interferon, cefprozil, ceftriaxone, cefoperazone-sulbactam, piperacillin– tazobactam, biapenem, meropenem, vancomycin, linezolid, sulfamethoxazole, levofloxacin moxifloxacin, glucocorticoids |  | (22) |
| Wen Wang | China | 2021 | January 21 to February 11 | Retrospective | 44 | 90/57 | 147 | 3 | lymphopenia and eosinopenia, Severe patients also had a higher level of D-dimer and CRP | fever, sputum cough | Not mention | Ribavirin, Lopinavir/Litonavir, Interferon, Oseltamivir, Antibiotic, Glucocorticoids, Intravenous immunoglobulin | 147/0 | (30) |
| Wei Wang | China | 2020 | February 9, 2020, and March 5, 2020 | Retrospective | 52 | 214/207 | 421 | 4 | Not mention | fever, cough, Sore throat | Secondary PTB | antibiotics, arbidol, chinese herbs | 362 Recover (4TB) | (31) |
| Xiaochen Li | China | 2020 | January 26, 2020, to February 5, 2020 | observational | 60 | 279/269 | 548 | 9  5 non-severe  4 severe | Increase: procalcitonin, globulin, LDH, NT proB-type natriuretic peptide, d-dimer, AAT, AST, total and conjugated bilirubin, blood urea nitrogen, and creatinine | fever, fatigue, sore throat, cough, chest pain, dyspnea |  | umifenovir, Oseltamivir, Lopinavir/ritonavir | 202(72.9%)/ 3 (1/1%) | (38) |
|  |  |  |  |  |  |  |  |  |  |  |  |  | 85(31.7 %)/ 87 (32.5%) |  |
| Nitin Goel | India | 2021 | June 17 2020 to October 22  2020 | Retrospective | 44.62 + 15.77 | 19/16 | 35 post covid | 5 | Increased: ALP, AST, ALT | breathlessness, fatigue, chest pain, cough | PTB  (Pan-susceptible  ) | Not mention | Not mention | (32) |
| R. Hesse | South Africa | 2020 | 1 March 2020 to 7 July 2020 | Retrospective | 42.3 | 37790/60545 | 98335 | 396 | critical group Increase: CRP, PCT, LDH, WCC, D-dimer and INR, RDW, troponin T  and NT-proBNP decrease: albumin, RCC, haemoglobin, haematocrit, MCHC | Not mention | PTB, TBM | Not mention | Not mention | (39) |
| LawrenceMwananyanda | Zambia | 2021 | June 15 and October 1, 2020 |  | 48 | 68/22 | 70 | 22 | Not mention | Not mention | Not mention | Not mention | 0/70 | (33) |
| James Mash | South Africa | 2020 | March _ June 2020 | Descriptive  observational | 46.3 | 571/805 | 1376 | 84 | Not mention | Cough, shortness breath, fever, body pains, myalgia and sore throat |  | Low molecular weight heparin ceftriaxone, azithromycin  or co-amoxiclav, proning or steroids | 525/12 | (85) |
|  |  |  |  |  |  |  |  |  |  |  |  |  | 147/19 |  |
|  |  |  |  |  |  |  |  |  |  |  |  |  | 130/73 |  |
|  |  |  |  |  |  |  |  |  |  |  |  |  | 7/47 |  |
| Claudia Stochino | Italy | 2020 | 3_28 March |  | 39 | 12/8 | 20 | 20 | Increased D-dimer, ferritin | Fever, cough, headache, chest pain | Not mention | - isoniazid, rifampicin, ethambutol and pyrazinamide , second-line: prothionamide, linezolid, terizidone clofazimine  - HCQ |  | (58) |

1. 48 patients had pulmonary TB (one caused by Mycobacterium bovis)

2. Of 49 patients, 26 (53.0%) had TB before COVID-19, 14 (28.5%) had COVID-19 first and nine (18.3%) had both diseases diagnosed within the same week (four on the same day). 26/49 (53.1%) patients were migrants, 15/48 (31.3%) unemployed, and 2/48 (4.1%) healthcare workers (medical doctor and radiology technician).

3. Out of 69 patients 43 were migrants

Table 2. COVID-19/TB case studies and case series information

| **Author** | **Country** | **Year** | **Type of study** | **Age (year)**  **Male/Female** | **Other Lab criteria** | **Clinical feature** | **Comorbidity** | **Ref.** |
| --- | --- | --- | --- | --- | --- | --- | --- | --- |
| M. Elziny | Qatar | 2021 | case study | 29/M | a white blood cell count of 3,000 cells/mm3, a C-reactive protein level of 252 mg/dL, and a ferritin level of 746 ug/L | 3-day fever and dry cough, 7 days abdominal distention | None | (60) |
| Stalz Charles Vilbrun | Haitian | 2020 | case study | 26/M | white blood cell count of 12,100 and a hemoglobin level of 9,600 g/dL., a creatinine level of 0.4 mg/dL, a blood urea nitrogen of 7 mg/dL, a glucose content of 154 mg/dL, and AST level of 110 U/L (normal range, 14–59) | Not mention | None | (87) |
| Bianchuan Cao | China | 2020 | case study | 47/F |  | Did not suffer from  fever, coughs, or difficulties in breathing | History of hemoptysis, asthma | (63) |
| Tekobo Abiodun Gbenga1 | Nigeria | 2020 | case series | 30/M | Blood pressure of 90/60 mmHg, oxygen saturation89%, leukocytosis with lymphopenia, anemia, thrombocytopenia | 6 months history cough productive of scanty whitish and occasional brownish sputum, no hemoptysis, low-grade fever, and weight loss | None | (53) |
|  |  |  |  | 33/M | Lymphopenia and anemia | 2 months cough, productive scanty whitish sputum, low-grade fever, and weight loss. | None |  |
| Todd Cutler | USA | 2020 | case study | 61/M | Lymphopenia | History of 4 days of cough and fever. acute high-grade fever and cough with a background of 4 months of cough with occasional blood-streaked sputum | Parkinson’s disease | (52) |
| Somasundram Pillay | South africa | 2021 | case study | 44/F | Not mention | 1-week poor appetite, night sweats, easy fatigability, non-productive cough, shortness of breath | HIV | (51) |
| C. LIU | China | 2020 | case series | 26/M |  | 2-week history of cough, shortness of breath and fever |  | (45) |
|  |  |  |  | 46/M |  | 13-day history of fever, myalgia (5 days before developing a fever, myalgia, sore throat, right chest pain, dyspnoea |  |  |
| José Arturo Martínez Orozco | Mexico | 2020 | case study | 51/M |  | anosmia, dysgeusia, and nocturnal diaphoresis. | type 2 diabetes mellitus 10 years previously | (79) |
| Farias | Brazil | 2020 | case series | 39/M | Low hemoglobin and hematocrit levels, lymphopenia, elevated CRP | 7-day fever, myalgia, headache, cough | HIV/ AIDS | (80) |
|  |  |  |  | 43/M | Low hemoglobin and hematocrit levels,  Elevated: Lactate dehydrogenase, CRP, D-dimer level (0.6 mcg/mL) exceeded the normal range | 1 month cough with hemoptoic sputum for, evolving to mild respiratory distress in the last 7 days. | HIV/ AIDS |  |
| Fah Bouaré | Marrakesh | 2020 | case study | 32/F | Anemia with 7.6 gram/deciliter hemoglobin, thrombocytopenia of 70,000 elements/ microliter, leucopenia of 2,880 elements/microliter, hyper-ferritinemia at 8,972 nanogram/milliliter. | fever, cough, headache, myalgia | HIV | (62) |
| Meng Tham | Singapore | 2020 | case series | 32/M |  | 2 days fever, cough | None | (50) |
|  |  |  |  | 33/M | lymphocytic exudative effusion with an adenosine deaminase (ADA) level of 130 U/L | 8 days fever, cough,1month weight loss | None |  |
|  |  |  |  | 22/M | lymphocytic exudative effusion with an ADA level of 112 U/L and interleukin-6 (IL-6) level of >1,000 pg/mL | 10 days fever and cough (associated with exertional dyspnea) and pleuritic chest pain. | None |  |
|  |  |  |  | 40/M | lymphocytic exudative effusion with an ADA level of 62 U/L and an IL-6 level of >1,000 pg/mL, | 3 days fever, cough | None |  |
| Goussard | South Africa | 2020 | case series | 3y and 8mo/F |  | Not mention | None | (19) |
| Neyla Rivas | Panama | 2020 | case series | 29/M | absence of leukocytosis, with mild neutrophilia, marked lymphopenia, and mild anemia  Elevated: CRP, ferritin, D-dimer, procalcitonin | 2-week nonproductive cough. dyspnea, asthenia, adynamia, and weight loss of about 30 pounds in the previous 5 months | HIV co-infection | (82) |
|  |  |  |  | 53/M |  | 7 days of unquantified fever, dyspnea exertion, asthenia, and adynamia | HIV co-infection |  |
| Farida Essajee | South Africa | 2020 | case study | 2y and 7mo /F | Elevated: white cell count, haemoglobin, platelets, CRP, Coagulation, fibrinogen, D-Dimer, ferritin | acute onset left-sided weakness and lethargy | None | (20) |
| Zhi Yao | China | 2020 | case series | 50/M | Elevated: ADA, LDH, Leukocyte, Neutrophil, Platelet, Fibrinogen, D-dimer, CRP, ESR.  Decrease: AST, Albumin, Hemoglobin, lymphocyte | fever and productive cough, Wheeze, Fatigue | None | (64) |
|  |  |  |  | 44/M | Elevated: CRP, ESR, Fibrinogen, D–dimer  Decrease: AST | fever, fatigue, Wheeze, headache, dry cough, Chills | None |  |
| Pinheiro | Brazil | 2020 | case study | 68/M | not mention | 1week dyspnea, fever, and cough | diabetic and hypertensive  and had chronic liver disease secondary to schistosomiasis | (81) |
| Shiun WoeiWong | Singapore | 2020 | case study | 47/M | Elevated: CRP | productive cough, pleuritic chest pain, fever for 2 days. | None | (88) |
| Sankalp Yadav | India | 2020 | case study | 43/M | low lymphocyte count  Elevated: CRP, LDH, ESR | 2 weeks cough with expectoration, chest pain, reduced appetite, fever with chills, and night sweats | None | (49) |
| ARIELLE SASSON | USA | 2020 | case study | 44/M | Elevated: CRP, LDH, fibrinogen, D-dimer, procalcitonin | dry cough and fever for 5 days. | diabetic | (65) |
| Sarah Ayad | USA | 2021 | case study | 58/M | Not mention | chest pain, myalgia, intermittent fevers, chills, and productive cough | None | (66) |
| Mohammed Khayat | Saudi Arabia | 2021 | case study | 40/F | Not mention | fever, cough and body aches | None | (89) |
| Baskara | Indonesia | 2020 | case study | 42/M | Elevated: random blood glucose, slightly neutrophilia | headache, shivering, cough with sputum, abdominal pain, and night sweats | diabetic | (48) |
| Guiqing He | China | 2020 | case series | 67/M | Lowest leukocyte and lymphocyte  Elevated: CRP, ESR, ALT | fever, dry cough, Chest tightness, dyspnea | None | (68) |
|  |  |  |  | 26/M | Lowest leukocyte and lymphocyte  Elevated: CRP, ALT | fever, dry cough, diarrhea | None |  |
|  |  |  |  | 76/M | Lowest leukocyte and lymphocyte Elevated: CRP, ESR, ALT | fever, dry cough, Chest pain, dyspnea | None |  |
| Fahad Faqihi | Saudi Arabia | 2020 | case study | 60/M | lymphocytopenia,  Elevated: CRP, LDH, ferritin | fever, persistent productive cough, chest pain, myalgias, fatigue and respiratory distress. | hypertensive and diabetic | (74) |
| Utpal Sarma | India | 2020 | case study | 53/F | Elevated: LDH, CRP, ferritin | 6 days breathing difficulty, fever, cough | Diabetes mellitus 2/chronic kidney disease | (67) |
| Maria Musso | Italy | 2021 | case study | 45/M | lymphopenia  Elevated: CRP, | cough, fatigue and weight loss since3 months before | None | (47) |
| Michelangelo Luciani1 | Italy | 2020 | case study | 32/F | lymphopenia  Elevated: CRP, procalcitonin, fibrinogen, d-dimer | 3-weeklong high fever and muscle pain | None | (75) |
| Shawn Gerstein | USA | 2021 | case study | 49/M | Elevated: CRP, D-dimer, Ferritin, LDH | two-week history of burning, diffuse abdominal pain radiating to his back, worsening abdominal distension, non-productive cough and orthopnea | None | (90) |
| Aqusa Zahid | Pakistan | 2020 | case study | 26/F | Not mention | fever, dry cough and hoarseness of voice for 1 month. | None | (91) |
| Rabia Can Sarinoglu | Turkey | 2020 | case series | 77/F | Elevated: CRP, LDH | high fever, respiratory difficulty and tachypnea diarrhea 15 day previously. One week later, sputum production was increased with high fever | hemodialysis patient  related with chronic kidney failure  HIV 1-2 Ab+Ag  EIA test found positive. HIV-1  viral load was 12,957 copies/mL | (78) |
|  |  |  |  | 39/F | low WBC count, lymphopenia  Elevated: CRP, LDH |  |  |  |
| Zohaib Yousafa | Qatar | 2020 | case series | 34/M | Not mention | Fever, productive cough, myalgias | None | (69) |
|  |  |  |  | 32/M |  | Dry cough, fatigue. | None |  |
|  |  |  |  | 50/M |  | Dry cough | Diabetes mellitus 2 |  |
|  |  |  |  | 35/M |  | dry cough, fever | None |  |
|  |  |  |  | 27/M |  | Fever, myalgias, headache | None |  |
|  |  |  |  | 35/M |  | Dry cough | None |  |
| Pierre Goussard | South Africa |  | case study | 2 Y 5M /M | white cell count of 8.03 × 109/L, neutrophils 3.56 × 109/L, lymphocytes 3.45 × 109/L, and C‐reactive protein of 11 mg/L. | fever, coughing, worsening respiratory distress, and loss of appetite | None | (21) |
| NATASHA GARG | USA | 2020 | case study | 44/M | leukocytosis  Elevated: CRP, LDH, ferritin, fibrinogen and IL6 | cough and fever of 5 days | hypertension, diabetes, atrial fibrillation | (42) |
| Abhijeet Singh | India | 2020 | case study | 47/F | Elevated: ESR, LDH, CRP, NT-pro Brain Natriuretic Peptide, ferritin, CPK and CPK-MB | 1.5 months history of low grade intermittent fever, non-productive cough and decreased appetite with an eventual weight loss of 4 kg. no prior history of pulmonary TB | None | (40) |
| Julien Lopinto | France | 2020 | case study | 58/M | CRP was measured at 256mg/ L, lactate dehydrogenase level was low 237 UI/ L and D-Dimer level was 1228 ng/ mL | fever, cough, increased expectoration, shortness of breath, myalgia and asthenia | None | (92) |
| Mihailo Stjepanović | Serbia | 2021 | case study | 27/M | Neutrophilia. Elevated: sedimentation rate, CRP, and presepsin | 3 days fever, fatigue and hemoptysis | None | (43) |
| Deepak Rajput | India | 2021 | case study | 21/F | Haemoglobin: 7.2 g/ dl, total leucocyte count: 16 109/L and platelet count: 99 109/L. | fever, cough, breathing difficulty | None | (93) |
| Mohammad H. AlKhateeb | Qatar | 2020 | case study | 28/M | vitamin B12 deficiency with iron stores depletion, Elevated: serum ferritin and D-dimer | six months history of dry cough and fever | None | (61) |

Table 2 continued. TB/COVID-19 case studies and case series information

| **Type of TB (Strain)** | **Treatment regimen (TB)** | **Treatment regimen (COVID)** | **COVID- 19 Severity rate / Recovery Time** | **Mortality** | **Ref.** |
| --- | --- | --- | --- | --- | --- |
| tuberculous peritonitis after COVID-19 millary TB  (susceptible-TB) | millary TB: ethambutol, isoniazid, rifampicin, pyrazinamide, amikacin for 10 days and piperacillin–tazobactam for 5 days | Oral azithromycin and HCQ | Hospitalization/ 36 day | No | (60) |
| MDR TB | Bedaquiline, levofloxacin, linezolid, clofazimine, pyrazinamide | Not mention | Hospitalization /3 weeks | No | (87) |
| secondary pulmonary TB | Isoniazid, rifampicin, ethambutol hydrochloride, and moxifloxacin hydrochloride | Lopinavir/ritonavi, change to umifenovir, twice daily | Hospitalization /14 days | No | (63) |
| MTB | anti-TB agent tabs pyridoxine, IV hydrocortisone | Lopinavir/ritonavir,azithromycin, Vitamin C, Zinc sulfate, oral prednisolone | Hospitalization /6 days | Yes | (53) |
|  | anti-TB agent, and tabs pyridoxine |  | Hospitalization /15 days | No |  |
| MTB | isoniazid, rifampin, ethambutol, and pyrazinamide | HCQ | Hospitalization | No | (52) |
| PTB | antituberculosis (Rifafour 3 tablets with pyridoxine) | Dexamethasone, vitamin D, zinc, thiamine, ascorbic acid and anticoagulation (enoxaparin 60 mg BD) |  | No | (51) |
| active PTB (MDR TB) | ethambutol, pyrazinamide, amikacin and levofloxacin | Arbidol, moxifloxacin, linezolid, cycloserine, clofazimine, and pyrazinamide | Hospitalization + oxygen therapy/2 weeks | No | (45) |
| pleural TB |  | Arbidol and moxifloxacin | Hospitalization + oxygen therapy/9 days | No |  |
| MBT (Sensitive to rifampicin) | Rifampicin, pyrazinamide, isoniazid, ethambutol | No safe or effective treatment just supportive care | 10 days | No | (79) |
| previous tuberculosis  (Susceptible TB) | Isoniazid, ethambutol, pyrazinamide, RIF initiated. He was also azithromycin (500 mg/day), HCQ (400 mg/5 days), and ceftriaxone (2 g/day). | Not mention | Not mention | No | (80) |
| Susceptible TB | isoniazid, ethambutol, pyrazinamide, RIF | Azithromycin, HCQ, ceftriaxone | Hospitalization /1 week | No |  |
| Lung miliary TB | Isoniazid, rifampin pyrazinamide, ethambutol | Chloroquine, azithromycin | Not mention | Low follow up | (62) |
| Not reported | anti TB | Not mention | Not mention |  | (50) |
| Not reported |  |  |  |  |  |
| Not reported |  |  |  |  |  |
| Not reported |  |  |  |  |  |
| PTB (susceptible TB) | Not mention | Not mention | Not mention | No | (19) |
| MTB (susceptible TB) | isoniazid, rifampin, ethambutol, pyrazinamide, intravenous levofloxacin azithromycin | HCQ orally, heparin | ICU | No | (82) |
| PTB (susceptible TB) | rifampicin/isoniazid | Ceftriaxone, azithromycin, enoxaparin, HCQ | Hospitalization | No |  |
| MTB (susceptible TB) | Isoniazid, rifampicin, pyrazinamide, ethionamide, prednisone, Aspirin | Not mention | Not mention | No | (20) |
| Active PTB | Anti TB | Not mention | Sever/22 days | yes | (64) |
| active PTB | isoniazid, rifampicin, pyrazinamide and ethambutol | Lopinavir/ritonavir | Moderate/26 days | No |  |
| MTB | Not mention | Not mention |  | No | (81) |
| Not reported | anti-TB | remdesivir | ICU/ after day 10 COVID-19 negative  after 2 weeks discharge | No | (88) |
| PTB (susceptible TB) | 4 antitubercular treatment | Not mention | Follow up / He has not yet reported back for follow-up | - | (49) |
| MTB (susceptible TB) | 4 anti-TB treatment | high dose steroids, Tocilizumab | Hospitalization | No | (65) |
| PTB | Not mention | Not mention |  |  | (66) |
| Latent TB change to active after covid19 treated (pan-sensitive) | Not mention | Not mention | Tell phone follow up/ symptom lasted 11 days | No | (89) |
| MTB (susceptible TB) | Isoniasid, Rifampicin, Pyrazinamide, Ethambutol | Oseltamivir, then given HCQ remaining days of treatment | after 13 days treatment | No | (48) |
| PTB | _ | Not mention | severe type / 37 days | No | (68) |
|  | _ |  | severe type /28 days | No |  |
|  | anti TB |  | severe type / 24 days | No |  |
| active TB (susceptible TB) | isoniazid (along with pyridoxal phosphate to avoid peripheral neuropathy), rifampicin, pyrazinamide, and ethambutol for two months, then isoniazid and rifampicin alone up to the present and for four months in total | Lopinavir/ritonavir and Ribavirin for 14-days, dexamethasone for 7 days, Prophylactic anticoagulation | ICU/ 27 days | No | (74) |
| PTB  (MDR TB) | anti TB | ampicillin, tab azithromycin, and vitamin C 500 mg | ICU / remain positive till the 44th day since the onset of symptoms | No | (67) |
| PTB (susceptible TB) | rifampicin, isoniazid, ethambutol pyrazinamide. Anti-TB regimen was modified for liver function impairment: rifampicin, ethambutol, amikacin and moxifloxacin | HCQ and corticosteroids | Dead after 7 days | yes | (47) |
| MTB | isoniazid, rifampicin, pyrazinamide, ethambutol | HCQ, lopinavir/ ritonavir | Hospitalization | No | (75) |
| extra-pulmonary tuberculosis | rifampin, isoniazid, ethambutol, and levofloxacin. Pyrazinamide was replaced by levofloxacin to decrease the hepatotoxicity risk | HCQ, convalescent plasma on hospital day 4 as part of a clinical trial, but did not meet criteria for remdesivir infusions | Hospitalization/6 weeks | No | (90) |
| MTB  (pan-sensitive TB) | Isoniazid, rifampicin, pyrazinamide and ethambutol | Not mention | MILD | No | (91) |
| Not reported | anti TB | Favipiravir, HCQ | Not mention | Not mention | (78) |
| Not reported | anti TB | Azithromycin, Plaquenil | Not mention | Not mention |  |
| PTB | Isoniazid, Rifampicin, Pyrazinamide, and Ethambutol with Pyridoxine. | Ceftriaxone, Azithromycin, and HCQ | 42 days | No | (69) |
| PTB |  |  | 42 days |  |  |
| PTB |  |  | 14 days |  |  |
| PTB |  |  | 72 days |  |  |
| PTB |  |  | 28 days |  |  |
| Pulmonary and pleural TB |  |  | 21 days |  |  |
| PTB (susceptible TB) | rifampicin, isoniazid, pyrazinamide, and ethambutol, adjuvant oral prednisone 2 mg/kg was added before the diagnosis of COVID‐19 | Not mention | 5 days | No | (21) |
| Not reported | rifampin, isoniazid, pyrimethamine and ethambutol | Plaquenil, ceftriaxone and azithromycin, dexamethasone 20mg IV daily for severe Acute respiratory distress syndrome (ARDS) | 51 days | No | (42) |
| PTB (rifampicin indeterminate) | rifampicin-R, isoniazid-Z, ethambutol-E and pyrazinamide-Z | azithromycin methylprednisolone, HCQ, vitamin C |  | No | (40) |
| post-tuberculosis bronchiectasis | Not mention | Not mention | ICU/14 days | No | (92) |
| TB | isoniazid, rifampicin, pyrazinamide, ethambutol | chloroquine and dual antibiotic therapy (fluoroquinolone and the third generation cephalosporine) | Hospitalization | No | (43) |
| Active TB | anti TB | Not mention | ICU | No | (93) |
| High resolution CT of the chest showed typical features for miliary TB | anti TB | Not mention | Not mention | Not mention | (61) |
